# Temporal bacterial and metabolic development of the preterm gut reveals specific signatures in health and disease

**DOI:** 10.1186/s40168-016-0216-8

**Published:** 2016-12-29

**Authors:** Christopher J. Stewart, Nicholas D. Embleton, Emma C. L. Marrs, Daniel P. Smith, Andrew Nelson, Bashir Abdulkadir, Tom Skeath, Joseph F. Petrosino, John D. Perry, Janet E. Berrington, Stephen P. Cummings

**Affiliations:** 1 Faculty of Health and Life Sciences, Northumbria University, Newcastle upon Tyne, NE1 8ST United Kingdom; 2 Department of Molecular Virology and Microbiology, Baylor College of Medicine, Alkek Center for Metagenomics and Microbiome Research, Houston, Texas 77030 USA; 3 Newcastle Neonatal Service, Royal Victoria Infirmary, Newcastle upon Tyne, NE1 4LP United Kingdom; 4 Department of Microbiology, Freeman Hospital, Newcastle upon Tyne, NE7 7DN United Kingdom; 5 School of Science and Engineering, Teesside University, Middlesbrough, TS1 3BX United Kingdom

**Keywords:** Preterm infant, Gut microbiome, Necrotising enterocolitis, Metabolomics

## Abstract

**Background:**

The preterm microbiome is crucial to gut health and may contribute to necrotising enterocolitis (NEC), which represents the most significant pathology affecting preterm infants. From a cohort of 318 infants, <32 weeks gestation, we selected 7 infants who developed NEC (defined rigorously) and 28 matched controls. We performed detailed temporal bacterial (*n* = 641) and metabolomic (*n* = 75) profiling of the gut microbiome throughout the disease.

**Results:**

A core community of *Klebsiella*, *Escherichia*, *Staphyloccocus*, and *Enterococcus* was present in all samples. Gut microbiota profiles grouped into six distinct clusters, termed preterm gut community types (PGCTs). Each PGCT reflected dominance by the core operational taxonomic units (OTUs), except of PGCT 6, which had high diversity and was dominant in bifidobacteria. While PGCTs 1–5 were present in infants prior to NEC diagnosis, PGCT 6 was comprised exclusively of healthy samples. NEC infants had significantly more PGCT transitions prior to diagnosis. Metabolomic profiling identified significant pathways associated with NEC onset, with metabolites involved in linoleate metabolism significantly associated with NEC diagnosis. Notably, metabolites associated with NEC were the lowest in PGCT 6.

**Conclusions:**

This is the first study to integrate sequence and metabolomic stool analysis in preterm neonates, demonstrating that NEC does not have a uniform microbial signature. However, a diverse gut microbiome with a high abundance of bifidobacteria may protect preterm infants from disease. These results may inform biomarker development and improve understanding of gut-mediated mechanisms of NEC.

**Electronic supplementary material:**

The online version of this article (doi:10.1186/s40168-016-0216-8) contains supplementary material, which is available to authorized users.

## Background

Survival after preterm birth is increasing, but this is associated with increased numbers of infants developing necrotising enterocolitis (NEC) and late onset sepsis (LOS), with ~10% and ~20% of very low birth weight (VLBW) infants being affected, respectively [1]. NEC and LOS are now responsible for more deaths after the first week of life in extremely preterm infants than any other single pathology [2], and both may result in significantly impaired outcomes in survivors. Antecedent risk factors for NEC vary, and the pathophysiology remains poorly defined. Currently accepted staging, e.g. Bell’s criteria, is deemed outdated [3] and there is increasing acceptance that a clinical diagnosis of “NEC” may simply be the final common pathway of multiple disease processes and LOS from gut bacterial translocation sharing similar processes [4].

Bacterial colonisation is necessary for the development of NEC, and specific bacterial taxa have been associated with onset, particularly those from the Proteobacteria phylum [5, 6] such as *Enterobacter* [7], *Escherichia* [8], *Sphingomonas* [9], and *Klebsiella* sp [10]. Studies from different research groups (and hence different neonatal intensive care units) identify a variety of signatures potentially associated with disease states. The two largest studies exploring NEC have shown conflicting findings, with no differences between NEC and control infants (*n* = 163 patients (21 with NEC), 482 samples) in a denaturing gradient gel electrophoresis (DGGE) and culture exploration [11] and more recently an increased relative abundance of Gammaproteobacteria and reduced Negativicutes (*n* = 166 patients, 3587 stools) [6]. Moving beyond 16S rRNA bacterial profiling, a metagenomic (shotgun sequencing) study of NEC samples during an apparent outbreak found no distinct microbial strain in NEC infants at the time of diagnosis [12]. Studies have also reported an absence of “pathogenic” bacteria in tissue resections from NEC infants [13] and no difference in the total bacterial load [14].

Previous studies utilising metabolomics to understand NEC pathogenesis and identify potential biomarkers are limited, with no published data on stool metabolomics. However, Morrow et al. (2003) coupled bacterial profiling of stool to metabolomic analysis of matched urine, showing early dysbiosis of the bacterial community and a high alanine to histidine ratio that was associated with microbial characteristics and good overall prediction of NEC [5]. Other metabolomic studies have utilised serum/plasma, employing either LCMS or gas chromatograph MS (GCMS), showing metabolomic profiles differ in disease compared to controls, but robust biomarkers were not found [15, 16].

To further progress understanding of the bacterial and host processes involved in NEC, we recruited a large cohort of preterm infants sampling stool daily where possible (*n* = >300 patients/>3000 samples) capturing key health related issues with precise definitions. Using strict classification for NEC and only including patients with robust temporal sampling before and after disease diagnosis, we present comprehensive longitudinal gut microbiome data on 641 samples from those with disease (*n* = 7) and well-matched non-diseased controls (*n* = 28). A subset of 75 samples (*n* = 16) also underwent ultra performance liquid chromatography mass spectrometry (UPLC-MS). We hypothesised that there would be detectable and coherent differences in bacterial and metabolomic profiles between healthy and diseased infants before the diagnosis of NEC, with associated bacterial signatures potentially predictive of health or disease onset.

## Results

### Bacterial Profiling

*Enterobacteriaceae*, *Escherichia*, *Enterococcus*, and *Staphylococcus* were present in every sample (core microbiome). The unclassified *Enterobacteriaceae* was cultured and identified by Matrix-assisted laser desorption/ionisation time-of-flight mass spectrometry (MALDI-TOF-MS) and full-length 16S sequencing as *Klebsiella* (*K. oxytoca* and *K. pneumoniae*). The developing gut microbiome in patients diagnosed with NEC was highly dynamic and individual, with no clear causative organism identified in the patients diagnosed with NEC (Additional file 1: Figure S1 and Additional file 2: Table S1). Comparably, the bacterial profiles from each control patient showed a high degree of intra- and inter-patient variability, with large temporal shifts in the core and satellite operational taxonomic units (OTUs) (Additional file 3: Figure S2).

**Fig. 1:**
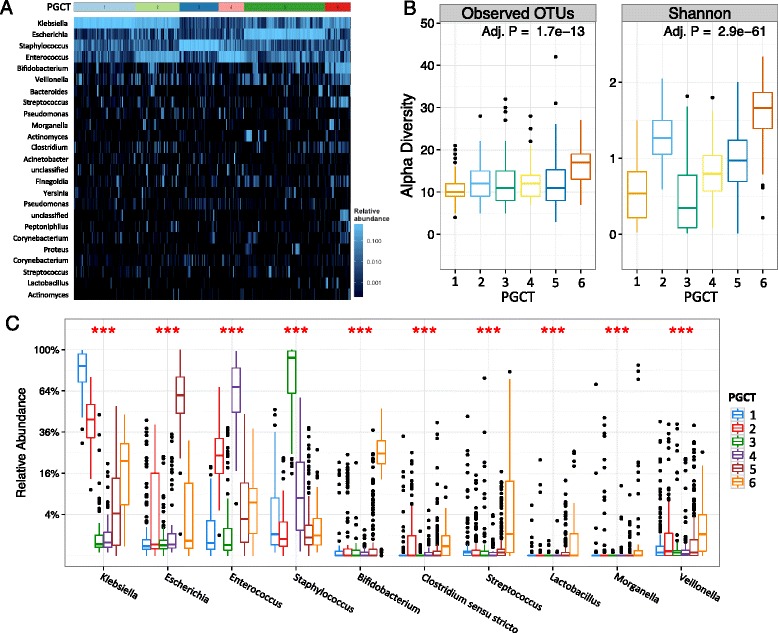
Analysis of each of the six preterm gut community types (PGCT). **a** Heatmap showing the six PGCTs and the status. Status based on disease or control; Control = *pink*, DControl = *green*, PreNEC = *blue*, and PreLOS = *purple*. Top 25 most abundant OTUs shown. **b** Boxplot analysis of the observed OTUs and Shannon diversity showing each PGCT. *P* values based on the Kruskal-Wallis test show a significant increase in number of OTUs and Shannon diversity in PGST 2. **c** Boxplot analysis of the eight most significantly distinct OTUs in each PGST. ***Represents a Kruskal-Wallis *P* value ≤ 0.001

To further explore the complexity in the developing preterm microbiome, we employed novel clustering analysis to ascertain preterm gut community types (PGCTs). Such approaches have been successfully applied to reduce the dimensionality of complex datasets, allowing robust analysis of temporal microbiome development and in determining the primary drivers of community structure (e.g. demographics, day of life, or disease) [17, 18]. We found all samples grouped into six discrete clusters (Fig. 1a; gap statistic Additional file 4: Figure S3). Each cluster, defined as a PGCT, was grouped by as follows: dominance of *Klebsiella* (PGCT 1), dominance of both *Klebsiella* and *Enterococcus* (PGCT 2), dominance of *Staphylococcus* (PGCT 3), dominance of *Enterococcus* (PGCT 4), dominance of *Escherichia* (PGCT 5), and mixed population with high relative abundance of *Bifidobacterium* (PGCT 6) (Fig. 1a; individual PGCT heatmaps Additional file 5: Figure S4). The number of observed OTUs was comparable between all PGCTs with the exception of PGCT 6, which had a significantly higher richness and diversity (*P* = >0.001) (Fig. 1b). Notably, this pattern in the Shannon diversity was uniform with increasing age (Additional file 6: Figure S5). Further analysis of the specific OTUs associated with each PGCT were in accordance with the heatmap analysis, with PGCTs 1, 3, 4 and 5 all characterised by significantly (*P* = <0.001) higher relative abundances of a single core OTU (Fig. 1c). PGCT 2 is further characterised by significantly (*P* = <0.001) greater relatively abundances of *two* core OTUs (*Klebsiella* and *Enterococcus*). PGCT 6 is uniquely characterised by the significantly (*P* = <0.001) high relative abundance of a *Bifidobacterium* OTU, as well as significantly (*P* = <0.001) higher relative abundances of *Clostridium* spp., *Streptococcus* spp., and *Lactobacillus* spp. (Fig. 1c).

**Fig. 2:**
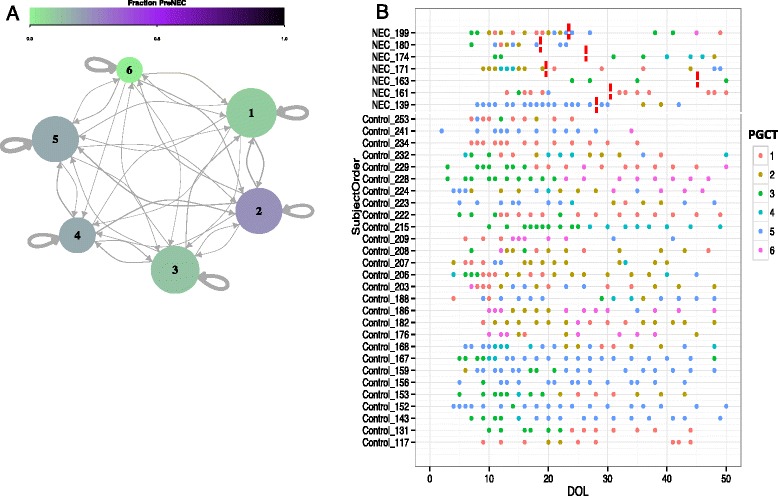
Dynamics of the microbiome through each preterm gut community type (PGCT) in patients diagnosed with NEC compared to matched controls over the initial weeks of life. **a** Transition network analysis showing PGCTs in the PreNEC samples. Approximated as a Markov chain with subject-independent transition probabilities. *Arrow weights* reflect the transition probabilities from the existing PGCT to the subsequent PGCT in next sample. Size of circle reflects the relative number of samples associated with that PGCT. Increasing fractions represent PGCT that have relatively larger number of predisease diagnosis samples. **b** Visualisation of the PGCTs in each individual patient overtime. *Red dotted lines* represent day of NEC diagnosis. Only samples up to day 50 of life are included. Patient 180 died during the study

Using network analysis with 10 days prior to disease diagnosis to classify samples as “PreNEC” (based on our previous work [19]), we were unable to confidently assign any PGCT to the PreNEC samples compared to all the control samples (Fig. 2a, b). PGCT 2 (fraction 0.16) and PGCT 5 (fraction 0.09) were most associated with subsequent NEC diagnosis (Fig. 2a). While no specific PGCT was associated with disease, PGCT 6 was not found in any sample from NEC patients prior to diagnosis. The only occurrence of PGCT 6 in a patient diagnosed with NEC occurred in patient 199 22 days following diagnosis (Fig. 2b). PGCT 6, which represents a diverse community high in relative *Bifidobacterium* abundance, occurred throughout the control population from early to late samples. While no single PGCT was specifically associated with NEC, the number of transitions between PGCTs was significantly increased (*P* = 0.043) prior to NEC diagnosis, compared to matched controls over the same timeframe. The temporal development of Shannon diversity increased in control infants, but reduced in the NEC samples (Additional file 7: Figure S6).

Interestingly, the relative abundance of *Staphylococcus* decreased in all groups following birth, with few samples containing a high relative abundance following day 25 of life. This was reflected in PGCT 3 (*Staphylococcus* dominated), which was the only PGCT associated with age. Additionally, no statistical difference in the relative abundance of any OTU was found between vaginal and caesarean section delivery from samples collected in the initial week of life. Similarly for the alpha diversity, while the Shannon diversity of vaginally delivered infants was higher than those delivered by caesarean section, this increase was not significant (Additional file 8: Figure S7). No significant difference was also found in the alpha diversity of the gut microbiome between infants born at different gestational ages (*P* = 0.16).

### Metabolite profiling

**Fig. 3:**
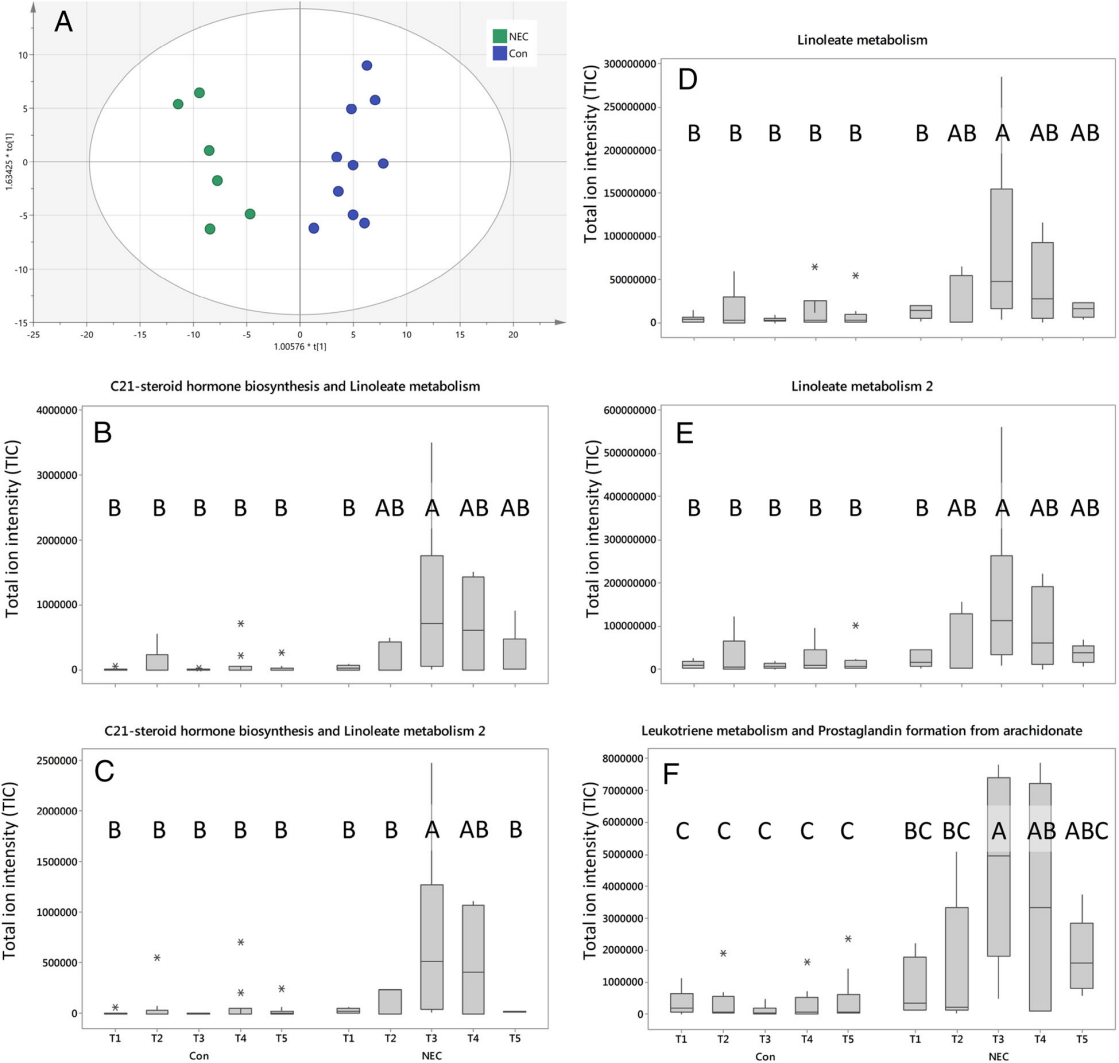
LCMS metabolomic features associated with NEC diagnosis. **a** OPLS-DA of NEC and matched control samples at time point 3 (diagnosis). **b**–**f** Box plot analysis showing the temporal development of the five most significant features associated with NEC diagnosis. *Lettering* represents Tukey’s pairwise comparison results, where groups that do not share a letter are significantly different. NEC diagnosis is significantly different from control samples. All time points included

**Fig. 4:**
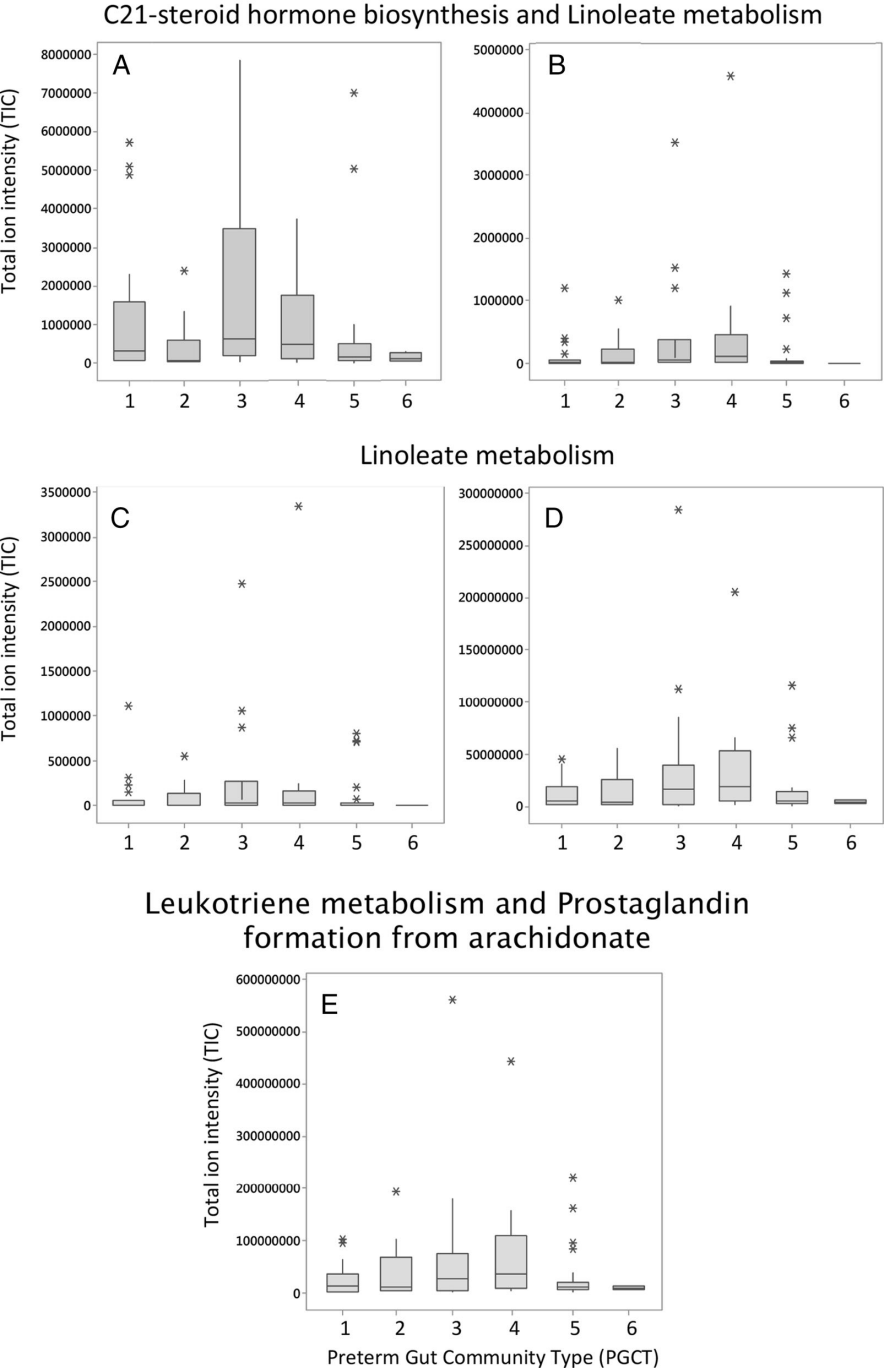
Comparison of significant LCMS metabolites associated with NEC and the PGCT determined by 16S bacterial profiling of the respective sample. Samples from all time points included in the analysis. **a**, **b** C21-steroid hormone biosyntehesis and linoleate metabolism pathways. **c**, **d** Linoleate metabolism pathways. **e** Leukotriene metabolism and prostagladlin formation from arachidonate pathway. PGCT 6 represents the community type associated with exclusively healthy samples

Mummichog analysis identified several pathways significantly associated with NEC. Orthogonal partial least squared-discriminatory analysis (OPLS-DA) analysis of all features identified as significant by Mummichog from samples at TP3 (disease diagnosis) showed clear clustering based on disease status (Fig. 3a). For NEC diagnosis the five features with the largest VIP scores and, thus most discriminatory power, corresponded to two metabolites from C21-steroid hormone biosynthesis and linoleate metabolism pathways, two metabolites from the linoleate metabolism pathway, and one metabolite from the leukotriene metabolism and prostaglandin formation from arachidonate pathway. Further analysis of these five features at all five time points showed significantly increased levels at diagnosis compared to all the control samples, with the total ion intensity increased through each time point prior to disease and reduced following diagnosis and treatment (Fig. 3b–f). The five most significant metabolites associated with NEC diagnosis were compared with each PGCT using data from all time points, showing clear concordance with the 16S bacterial profiling data (Fig. 4). The features most associated with NEC were reduced in relative abundance in PGCT 6.

## Discussion

The extensive longitudinal sampling employed in this study demonstrated the extent of intra- and inter-individual variation in the development of the preterm gut microbiota in both health and disease. To facilitate comparisons between samples prior to disease diagnosis and from healthy (control) infants, we clustered samples into one of six specific PGCT based on the bacterial profiles. The PGCT analysis showed the majority of patients exhibited multiple PGCTs during the study, with only one patient (control 234) having the same PGCT (PGCT 1) throughout the entirety of sampling. Indeed, controls generally showed significantly increased stability compared to NEC infants prior to disease diagnosis.

No specific microbial signature was found prior to the diagnosis of NEC, compared to matched healthy controls. Although no microorganism has been reproducibly associated with NEC, many studies have reported potential associations with specific taxa [7, 9, 10, 20, 21], including a recent large multicentre study which found increased Gammaproteobacteria and Negativicutes prior to NEC [6]. The latter study conducted analysis at the class level, whereas the current study used genus level taxonomic classification. Notably, analysing the current dataset at class level showed no significant difference in the relative abundance of Gammaproteobacteria (class) between the preNEC and control samples (Additional file 9: Figure S8). It should also be noted that by nature of “association” between specific taxa and NEC, the reported taxa are not ubiquitously increased in NEC and will also be present in comparable abundance in some healthy controls, which is in accordance with our data. Thus, the presence of such taxa alone may not improve our understanding of NEC pathogenesis. Our study is also consistent with another large preterm cohort (163 infants, 21 with NEC, analysing 482 samples) who report no associations using molecular DGGE profiling [11] and a recent powerful metagenomic study with analysis to the strain level [12, 22]. The latter metagenomic study highlights the importance of lower taxonomic level analysis in disease association studies. Further characterisation of *Escherichia coli* isolates from NEC and control infants in this study support the finding that each infant harboured unique strains (Additional file 10: Figure S9). While no specific microbial signature or taxa was evident in disease, the instability of the developing gut microbiome in NEC infants may be a “trigger” initiating and promoting a constant exaggerated inflammatory response, ultimately leading to tissue damage. Such findings need to be validated and quantified in independent cohorts, using rigorous longitudinal sampling prior to disease and similarly in controls.

Although no specific taxa was elucidated in bacterial profiling, metabolomic profiling showed that the five most discriminatory metabolites in the NEC samples at the time of diagnosis were significantly elevated compared to all the control samples. While the metabolites were most significant at diagnosis, the metabolites increased in intensity prior to diagnosis and reduced following NEC diagnosis. The temporal increase in metabolites prior to diagnosis, whilst levels in the control samples remained consistently low, suggests such metabolites may be predictive of disease onset. This finding offers potential in biomarker development for detecting the risk NEC, which precedes the clinical diagnosis. Based on the findings here, the progression toward NEC may be detected between 1–2 weeks prior to current clinical diagnosis, which is consistent with the previous data [22]. Such findings require validation in large multicentre studies, but the finding that 4/5 metabolites most associated with NEC were involved in linoleate metabolism is intriguing given that *trans*-10, *cis*-12-conjugated linoleic acid (*t*10*c*12-CLA) negatively impacts on lipid metabolism, mediated by alterations in gut microbiome [23]. As more metabolomic investigations are performed, the mechanism of linoleate metabolism in an inflammatory-mediated damage may be better elucidated, but the results of this study support the concept that it is, at least in part, mediated by the microbiome.

Analysing a large number of samples from healthy control infants revealed a novel healthy microbial signature (PGCT 6), characterised by dominance of bifidobacteria and a significantly higher overall diversity. In accordance with existing data, we found that control infants have increasing diversity whereas patients diagnosed with NEC have significantly reduced diversity while on the NICU [10, 19, 20], although the microbiome of NEC infants converge to that of controls within 1 year following NICU discharge [24]. As well as bifidobacteria, PGCT 6 also had significantly higher relative abundance of lactobacilli. Interestingly, because of the associated health benefits, these genera are the most common bacteria used in manufactured probiotic products [25]. The metabolomic profiling showed high concordance with the 16S bacterial profiling results. Strikingly, the metabolites most associated with NEC were significantly reduced in PGST 6 and the metabolites most associated with control were increased in PGST 6. This suggests that changes in the microbial community have a direct impact on the metabolic functioning on the host, which may ultimately contribute to disease pathophysiology.

Despite the recent trial showing no effect of supplemental *Bifidobacterium breve* [26], probiotics containing *Bifidobacterium* spp. have generally shown reduced incidences of NEC [27, 28] and bifidobacteria are known to play fundamental roles in the development and maintenance of a healthy gut microbiome [29, 30]. Thus, the high abundance of bifidobacteria may contribute to the improved health status by aiding gut maturation and preventing over-expression of potentially damaging pathways, although it is also possible that a higher abundance of bifidobacteria simply acts as a marker for a more healthy gut, rather than being directly causative in preterm infants. This is supported by the finding in this study that prostaglandin formation is significantly increased at NEC diagnosis and reduced in PGCT 6, with this pathway known to mediate inflammation [31, 32]. Caesarean or vaginal delivery did not significantly alter the preterm gut microbiome, even in the initial weeks of life, although PGCT 6 did occur more frequently in vaginally delivered infants compared to caesarean section (42 vs. 25, respectively).

Despite the collection of >3000 samples from >300 infants, the number of infants diagnosed with a disease from this cohort remains small due to both application of a strict definition of disease states and the inclusion criteria requiring robust temporal sampling prior to and following disease diagnosis. The extent of inter-individual microbial variability in both healthy and diseased infants suggests that no specific bacterial signature is likely to cause NEC in our unit, and this finding is unlikely to be influenced by additional cases of NEC.

## Conclusions

No consistent microbial signature or taxa was associated with NEC, but the number of PGCT transitions, and thus microbiome instability, was significantly increased in NEC and may contribute to, or be a marker of, the inflammatory-mediated damage in NEC pathogenesis. A novel diverse bacterial community with high relative abundance of bifidobacteria was found only in healthy samples. The metabolomics and 16S data was highly concordant, where metabolites associated with NEC were significantly reduced in the healthy PGCT. Strategies aiming to improve outcomes in preterm infants which focus on attempts to promote healthy PGCT profiles, rather than focusing on PGCTs potentially associated with later disease, are warranted.

## Methods

### Participants and study design

All infants were cared for in the neonatal intensive care unit (NICU) of the Royal Victoria Infirmary. Standardised feeding, antibiotic and antifungal guidelines were used as described previously [19]. All samples were collected before any probiotic was used on the unit. Stool samples and clinical data were collected from 318 preterm infants. NEC was categorized by at least one senior clinician and two additional senior research clinicians by conducting case reviews, reviewing notes, X-ray and operative findings and classified as either surgical or medical (no surgery). Complete agreement and presence of unequivocal radiographic signs (pneumatosis) was required for medical cases. All demographic information is summarised in Table 1, and full demographic information for each patient is presented in Additional file 11: Table S2.

**Table 1:** Summary of patient samples and demographic per group

	Control (*n* = 28)	NEC (*n* = 7)	*P* value
Number of stool samples	520	121	–
Gestation (weeks)^a^	27 (24–30)	26 (23–30)	0.599
Birth weight (g)^a^	910 (545–1810)	760 (500–1470)	0.416
Birth mode (CS/vaginal)	15/13	3/4	1.0
Gender (male/female)	20/8	3/4	0.345
Antibiotic prediagnosis (days)^a^	–	14 (2–26)	–
Antibiotic total (days)^a^	4.5 (2–31)	29 (6–44)	0.0002

^a^Median (range)

Seven well-sampled cases of NEC were selected and each matched to four well-sampled controls based on gestational age (GA; +/− 1 week), birth weight, and delivery mode. The 35 infants contributed a total 641 analysed stool samples. Samples were stored immediately at −20 **°**C and long-term at −80 **°**C, with all samples having comparable collection/storage. 16S rRNA gene bacterial profiling was performed on all the samples in the study. Metabolomic profiling was performed on a subset of 16 infants (75 stools), 6 NEC and 10 matched controls. Samples were selected for analysis relative to disease diagnosis at day of life (DOL) −14 (time point 1; TP1), −7 (TP2), 0 (TP3), +7 (TP4), and +14 (TP5), and matched by DOL alone in controls.

### 16S rRNA gene Bacterial Profiling

Nucleic acid extraction of stool was carried out on 100 mg of sample using the PowerLyzer™ PowerSoil® DNA Isolation Kit (MoBio, CA, USA) in accordance with the manufacturer’s instructions. Bacterial profiling utilised the 16S rRNA gene targeting variable region four based on the Schloss wet-lab MiSeq SOP and resulting raw fastq data were processed using Mothur (version 1.31.2) as described previously [33]. Briefly, combined reads were trimmed to 275 reads with 0 ambiguous bases. Chimeric sequences were detected by Chimera.uchime and removed from downstream analysis. Alignment was generated via the Silva v4 database [34] and Chloroplast, Mitochondria, unknown, Archaea, and Eukaryota linages were removed from the analysis. In total, 44,515,418 reads were obtained and raw sequences were deposited in MG-RAST under the accession numbers 4516545.3-4516585.3.

### UPLC-MS metabolomic profiling

Metabolomic profiling was performed as previously described [24]. Briefly, 100 mg stool was homogenised (80% methanol), vortexed for 15 min, centrifuged (10,000 × *g*), and lyophilised. Reverse-phase ultra-performance LCMS tandem mass spectrometry (UPLC-MS/MS) was performed using an Accucore C18 column (2.6 μm, 150 × 2.1 mm) at 40 °C, 3.0 μl injection, and 300 μl/min flow rate. Gradients increased from 5% acetronitrile (ACN) to 95% ACN over 22 min, followed by 8 min wash and re-equilibration. Samples were run randomly in triplicate on a Q-Exactive (Thermo) using HESI with high resolution (70,000) positive and negative switching. The mass range was set from 100–1000 m/z. SIEVE (Version 2.2 *beta*) was used to process the Thermo RAW files by component extraction detecting a total of 11,612 components (8343 positive and 3269 negative).

### Bioinformatic and statistical analysis

Analysis and visualization of microbiome communities was conducted in R [35], utilizing the phyloseq package [36] to import sample data and calculate alpha- and beta-diversity metrics. Each sample was rarefied to 4397 reads. Significance of categorical variables were determined using the non-parametric Mann-Whitney test for two category comparisons or the Kruskal-Wallis test when comparing three or more categories. All *p* values were adjusted for multiple comparisons with the FDR algorithm.

An R script was implemented and made publicly available by DiGiulio et al. [17] was employed for linear mixed-effects modelling, partition around medoid based clustering, and Markov chain modelling to cluster all the samples within the study into PGCTs and determine temporal development in health and disease. Bray-Curtis was used to calculate the distance between all the samples, and this was denoised by extraction of the most significant principal coordinates analysis (PCoA) eigenvectors before applying the PAM algorithm. Gap statistic was used to determine the number of clusters. Ten days prior to diagnosis of disease, network analysis was used to classify PreNEC, based on previous data [19]. To determine if the stability was potentially contributing to the disease pathophysiology, the total number of unique PGCTs and the number of transitions between PGCTs were compared for NEC patients, to matched controls. Only the pre diagnosis samples and control samples in the first 28 days of life were included (last predisease sample was day of life 28).

UPLC-MS data was filtered to include only *m/z* features that occurred in >20% of samples. Metabolite annotation and pathway enrichment was performed using Mummichog [37]. Mummichog was used to determine significant pathways at TP3 by comparing NEC vs. control. Only pathways identified in Mummichog were included in subsequent analysis. SIMCA 13.0 (Umetrics, Stockholm, Sweden) was used to model the metabolomics dataset by first plotting an unsupervised principal coordinate analysis (PCA) and if clear grouping was apparent a orthogonal partial least squared-discriminatory analysis (OPLS-DA) was performed. Only OPLS-DA with *R*2 > 0.8 and *Q*2 > 0.5 were considered robust and predictive, respectively. The variable importance plot (VIP) was used to determine the five metabolites most associated with each class [38]. MiniTab 17 was used to generate box plots and determine the significance by Tukey’s method for multiple comparisons.

## Additional files

Additional file 1: Figure S1. Area charts of infants diagnosed with NEC based on all OTUs. Legend restricted to 8 OTUs for clarity.*Dashed red lines* indicate day of NEC diagnosis and *dashed black lines* represent start of antibiotic course. Patient 180 died during the study. (PDF 768 kb)
Additional file 2: Table S1. Table of antibiotic information for patients diagnosed with NEC. (DOCX 21 kb)
Additional file 3: Figure S2. Longitudinal development of the core (*dashed lines*) and satellite (*solid lines*) communities in each control infant. Core OTUs defined as OTUs present in every sample. Satellite OTUs are the remaining OTUs with the highest overall relative abundance. (PDF 150 kb)
Additional file 4: Figure S3. Gap statistic to determine the number of clusters in the dataset. Based on this figure a K of 6 was chosen for this study, which corresponds to the point where the slope begins to plateau. (PDF 30 kb)
Additional file 5: Figure S4. Each individual heatmap and the corresponding preterm gut community type (PGCT). **A**) PGCT 1. **B**) PGCT 2. **C**) PGCT 3. **D**) PGCT 4. **E**) PGCT 5. **F**) PGCT 6. (PDF 460 kb)
Additional file 6: Figure S5. Shannon diversity indices overtime of all samples grouped according to preterm community type (PGCT). Grey shading indicates the 95% confidence interval of the mean. (PDF 64 kb)
Additional file 7: Figure S6. Shannon diversity indices overtime of all samples grouped according to patient status. Grey shading indicates the 95% confidence interval of the mean. (PDF 40 kb)
Additional file 8: Figure S7. Shannon diversity indices overtime in infants born by vaginal (V) or caesarean section (CS) delivery. Grey shading indicates the 95% confidence interval of the mean. (PDF 44 kb)
Additional file 9: Figure S8. Comparison of the relative abundance of Gammaproteobacteria between preNEC and controls. Only the preNEC samples included from NEC infants and only samples up to day 28 of life in controls. **A**) Regression analysis over day of life (DOL). **B**) Box plots. (PDF 52 kb)
Additional file 10: Figure S9. Pulse field gel electrophoresis (PFGE) of two *E. coli* isolates from NEC patients and 4 *E. coli* isolates from control patients. Two isolates from samples 1376 (patient 178) were analysed for confirmatory purposes. (TIF 655 kb)
Additional file 11: Table S2. Full demographics per patient. (XLSX 49 kb)

## Abbreviations

LOS: Late onset sepsis
NEC: Necrotising enterocolitis
NICU: Neonatal intensive care unit
PGCT: Preterm gut community type
UPLC-MS/MS: Ultra-performance liquid chromatography mass spectrometry tandem mass spectrometry
