# Supplementary figures and images for: Temporal bacterial and metabolic development of the preterm gut reveals specific signatures in health and disease
Source: Microbiome. 2016 Dec 29;4:67. doi: 10.1186/s40168-016-0216-8 (PMC5200962; doi:10.1186/s40168-016-0216-8)

Relative abundance

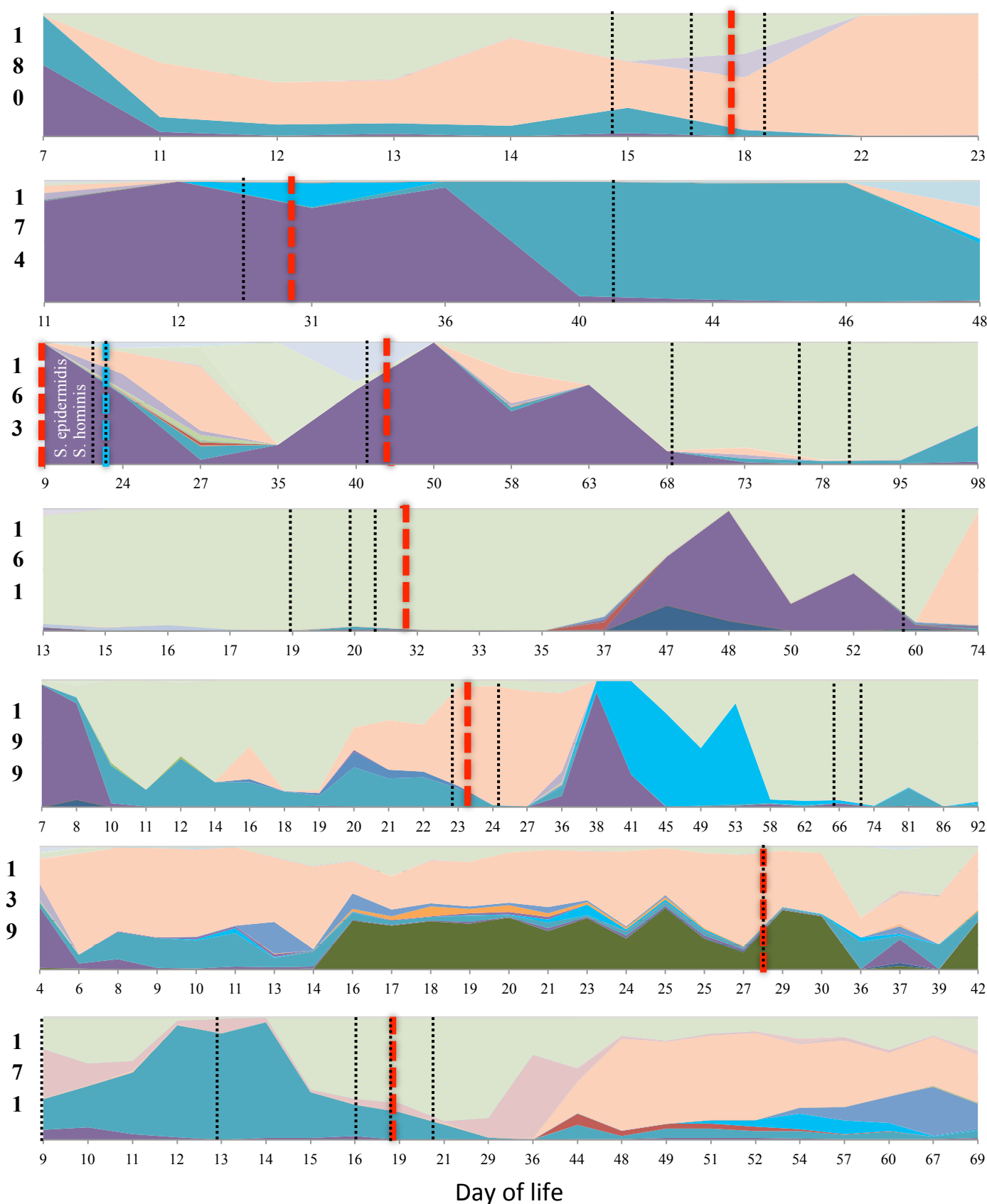

Supplement: Additional file 1: Figure S1. — Area charts of infants diagnosed with NEC based on all OTUs. Legend restricted to 8 OTUs for clarity.Dashed red lines indicate day of NEC diagnosis and dashed black lines represent start of antibiotic course. Patient 180 died during the study. (PDF 768 kb) [file 40168_2016_216_MOESM1_ESM.pdf]

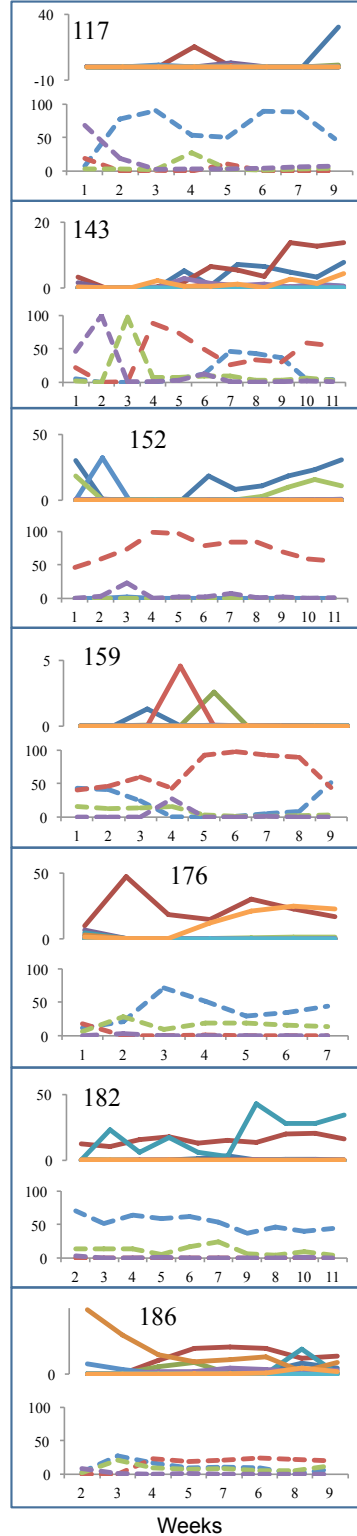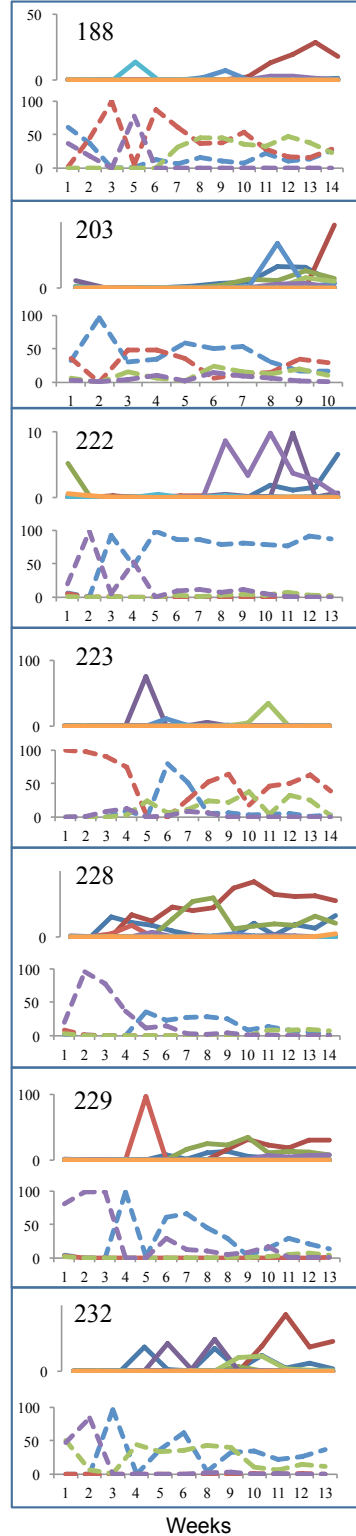

## Satellite OTUs

### Core OTUs

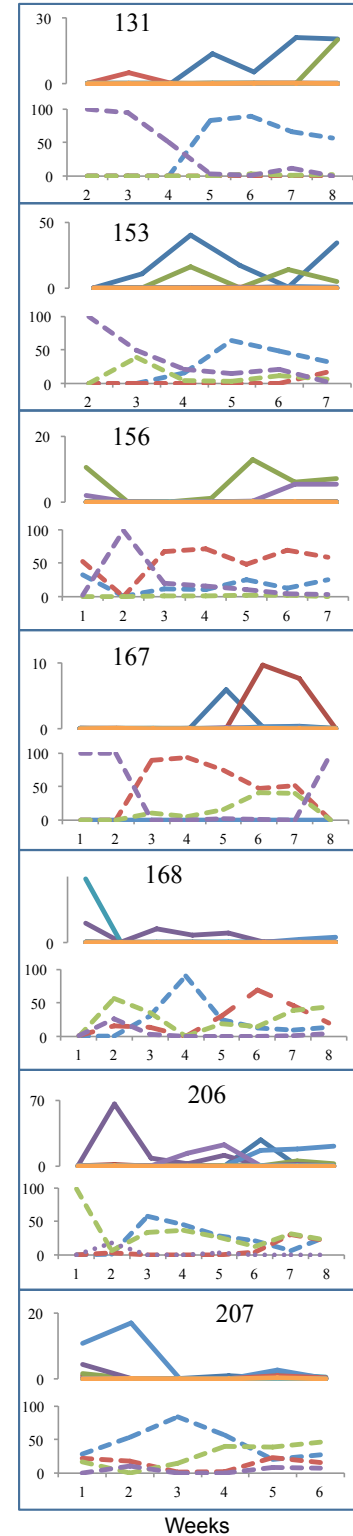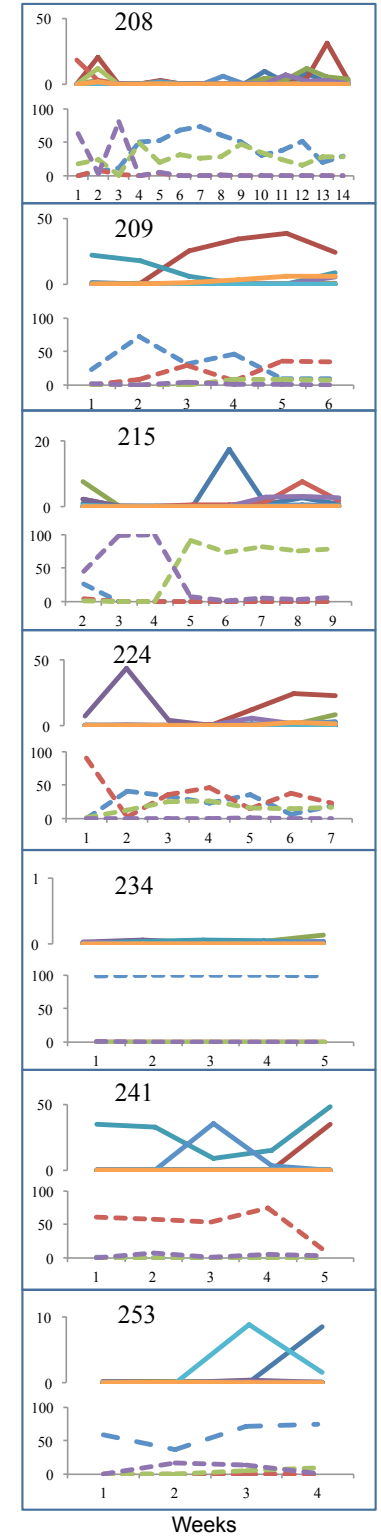

Supplement: Additional file 3: Figure S2. — Longitudinal development of the core (dashed lines) and satellite (solid lines) communities in each control infant. Core OTUs defined as OTUs present in every sample. Satellite OTUs are the remaining OTUs with the highest overall relative abundance. (PDF 150 kb) [file 40168_2016_216_MOESM3_ESM.pdf]

Gap Statistic results

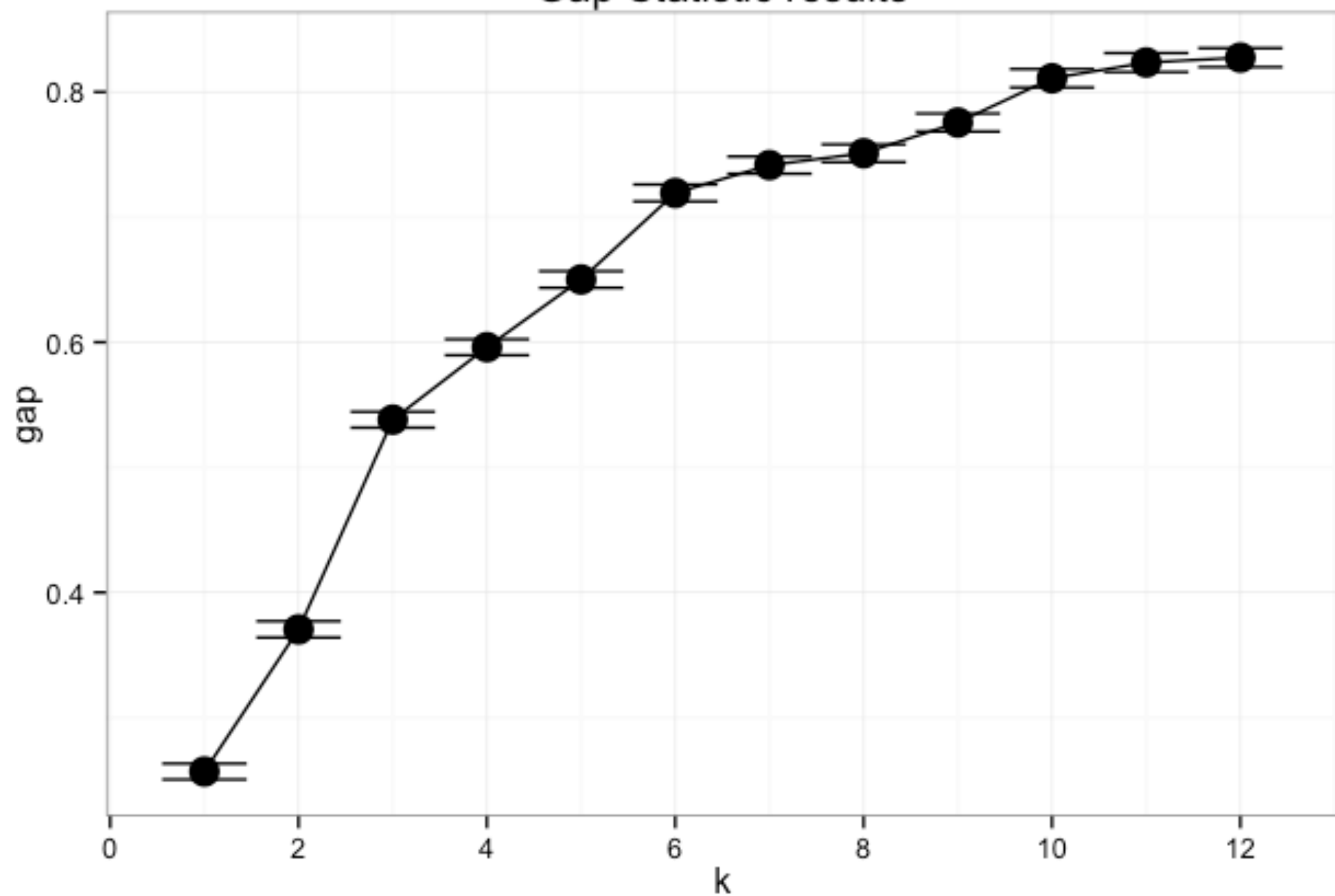

Supplement: Additional file 4: Figure S3. — Gap statistic to determine the number of clusters in the dataset. Based on this figure a K of 6 was chosen for this study, which corresponds to the point where the slope begins to plateau. (PDF 30 kb) [file 40168_2016_216_MOESM4_ESM.pdf]

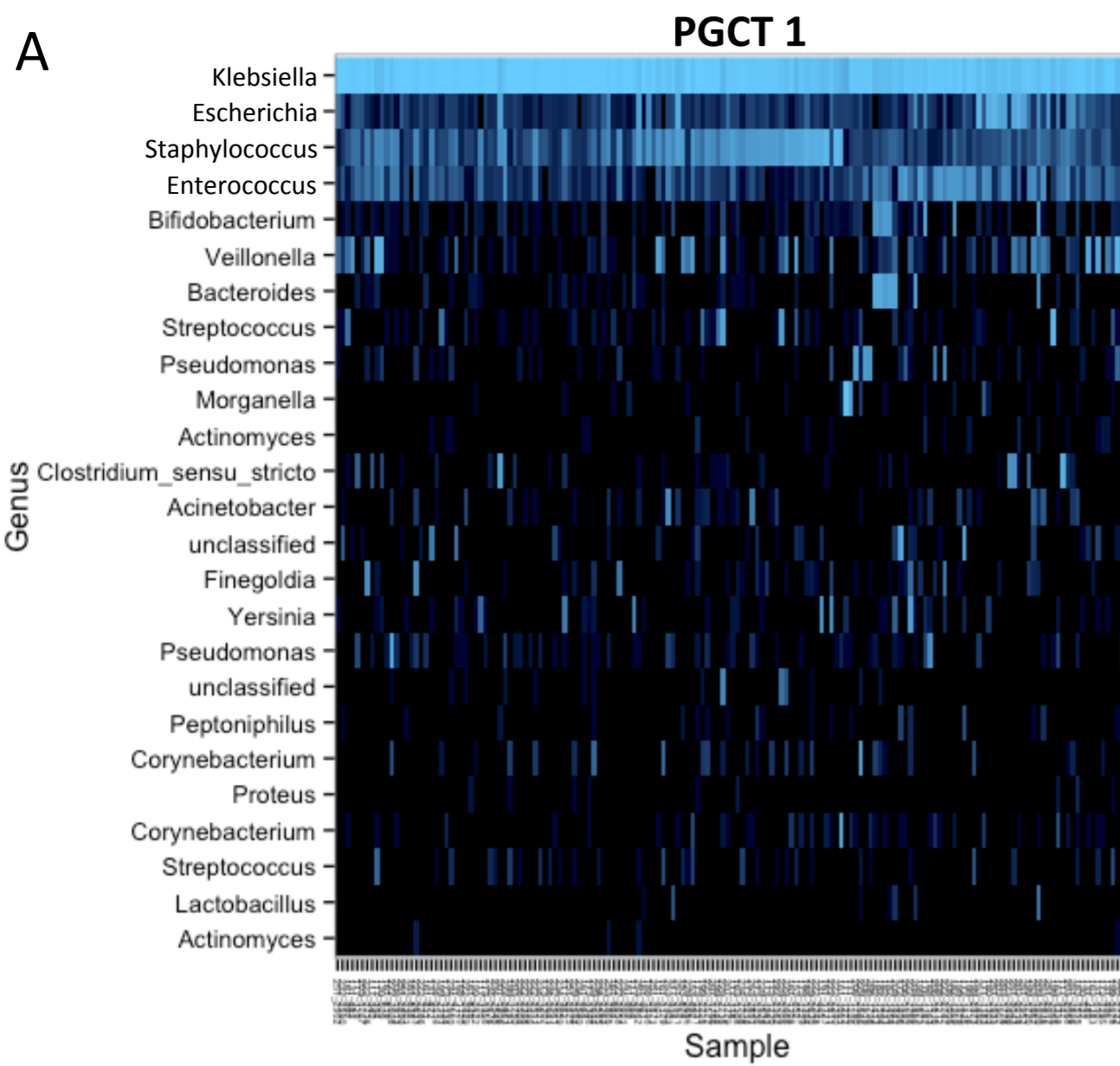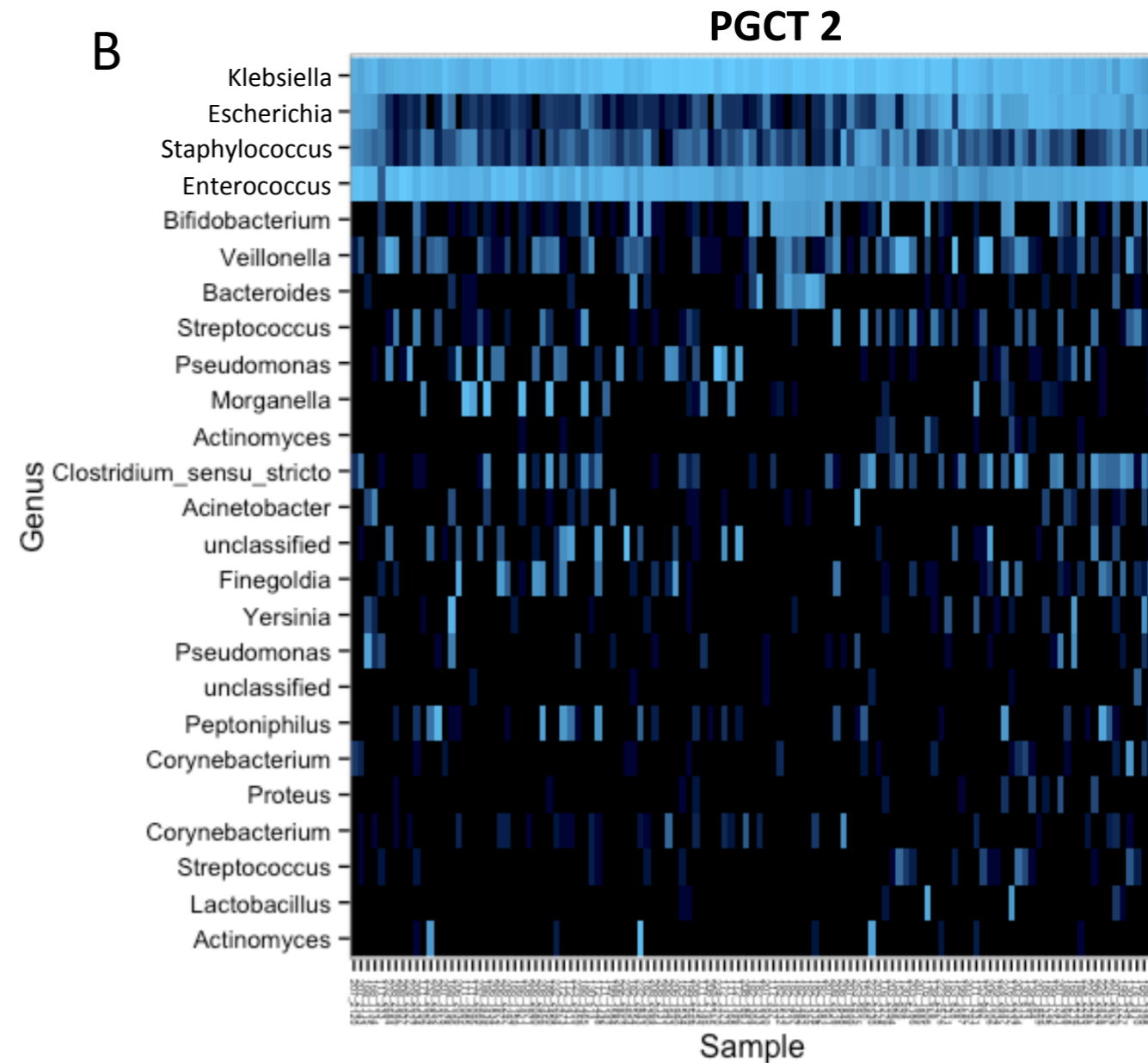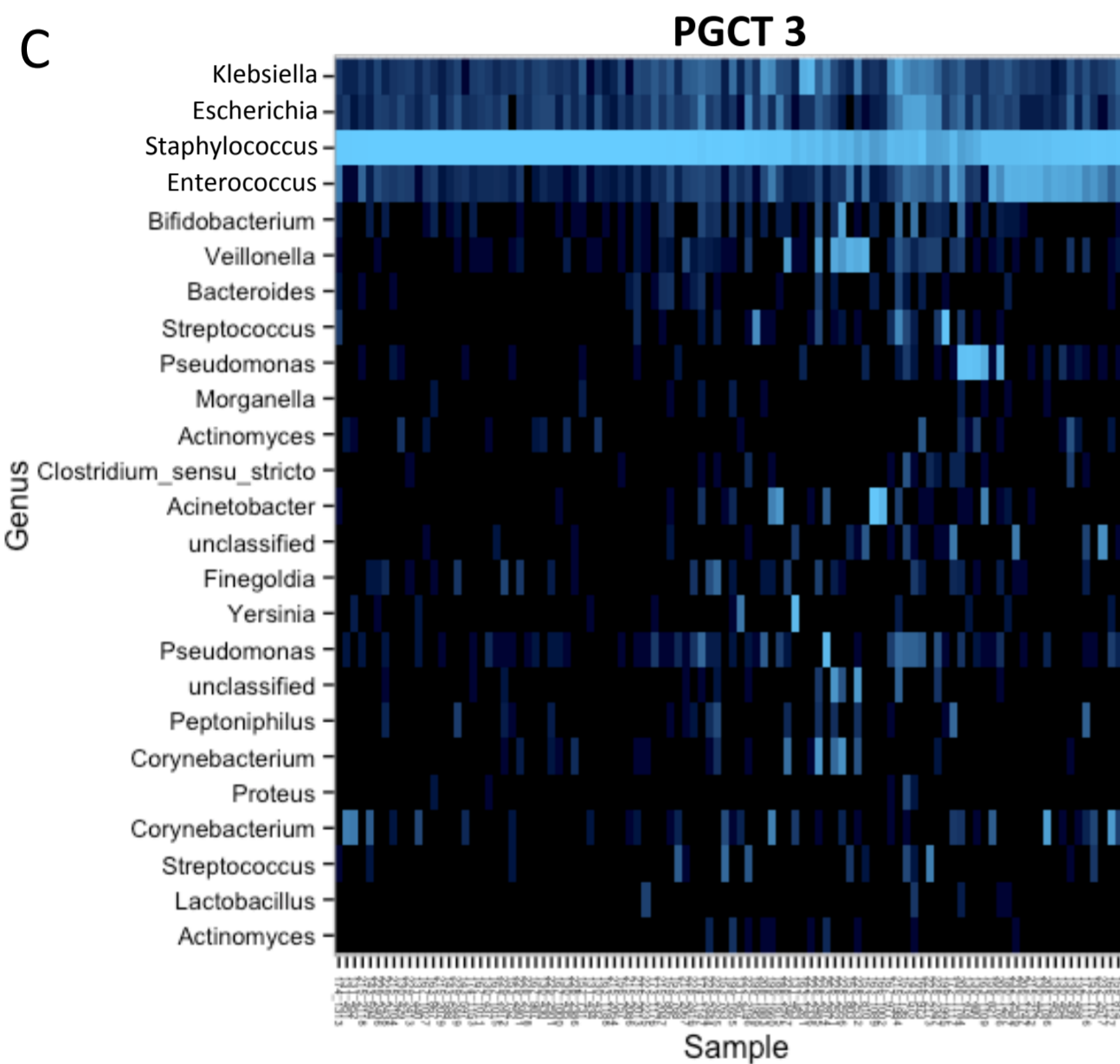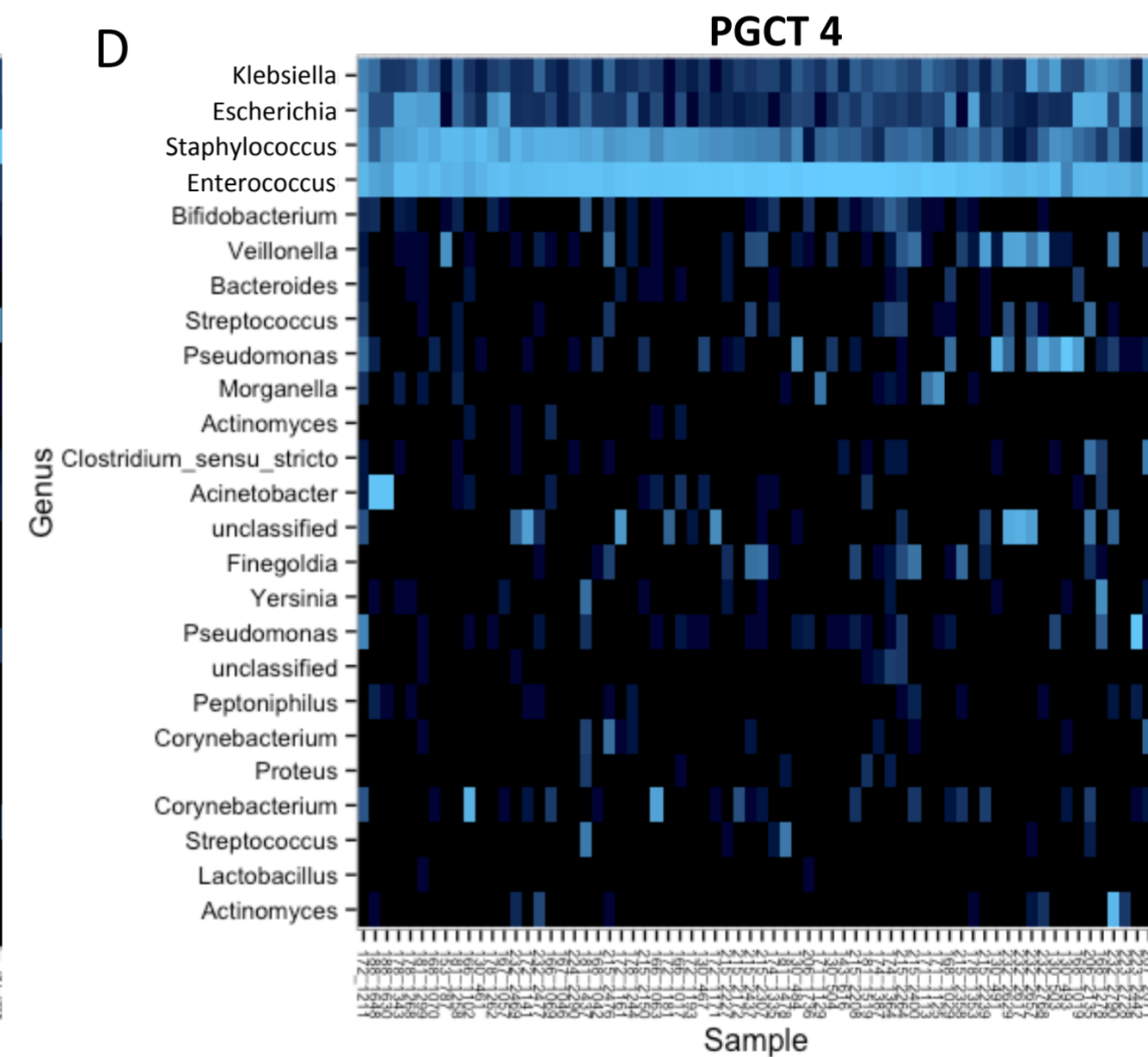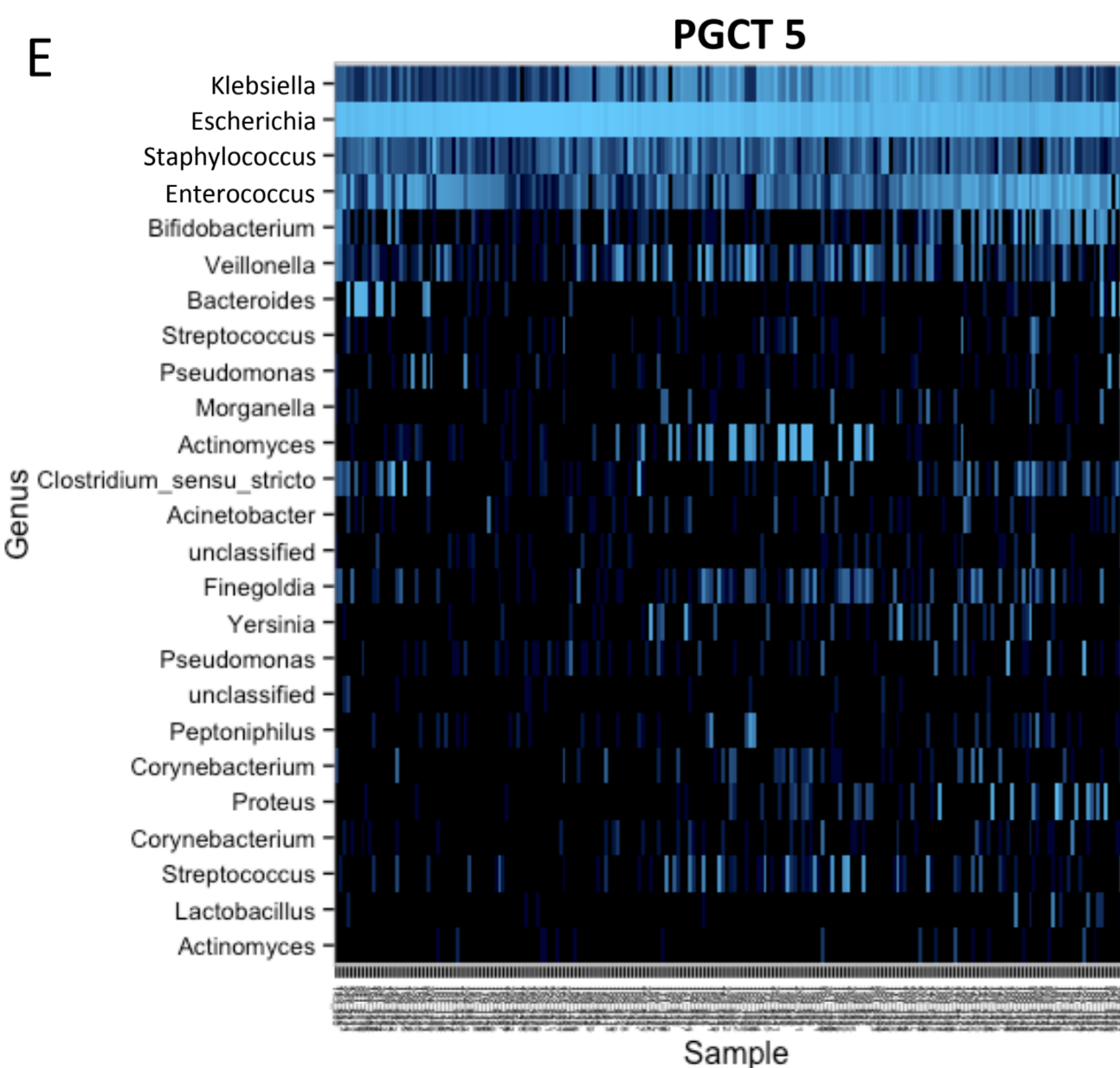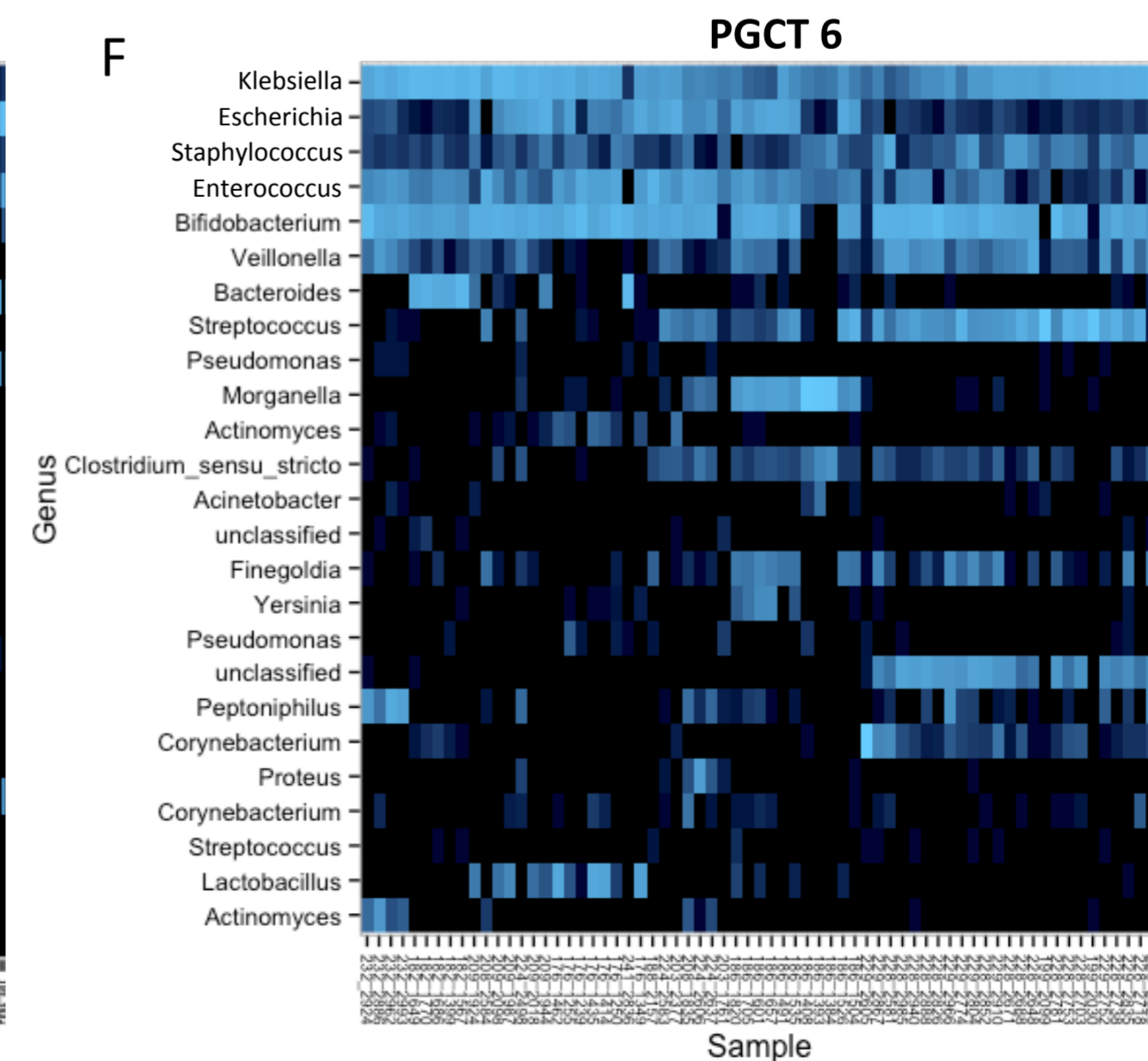

Supplement: Additional file 5: Figure S4. — Each individual heatmap and the corresponding preterm gut community type (PGCT). A) PGCT 1. B) PGCT 2. C) PGCT 3. D) PGCT 4. E) PGCT 5. F) PGCT 6. (PDF 460 kb) [file 40168_2016_216_MOESM5_ESM.pdf]

Shannon Diversity

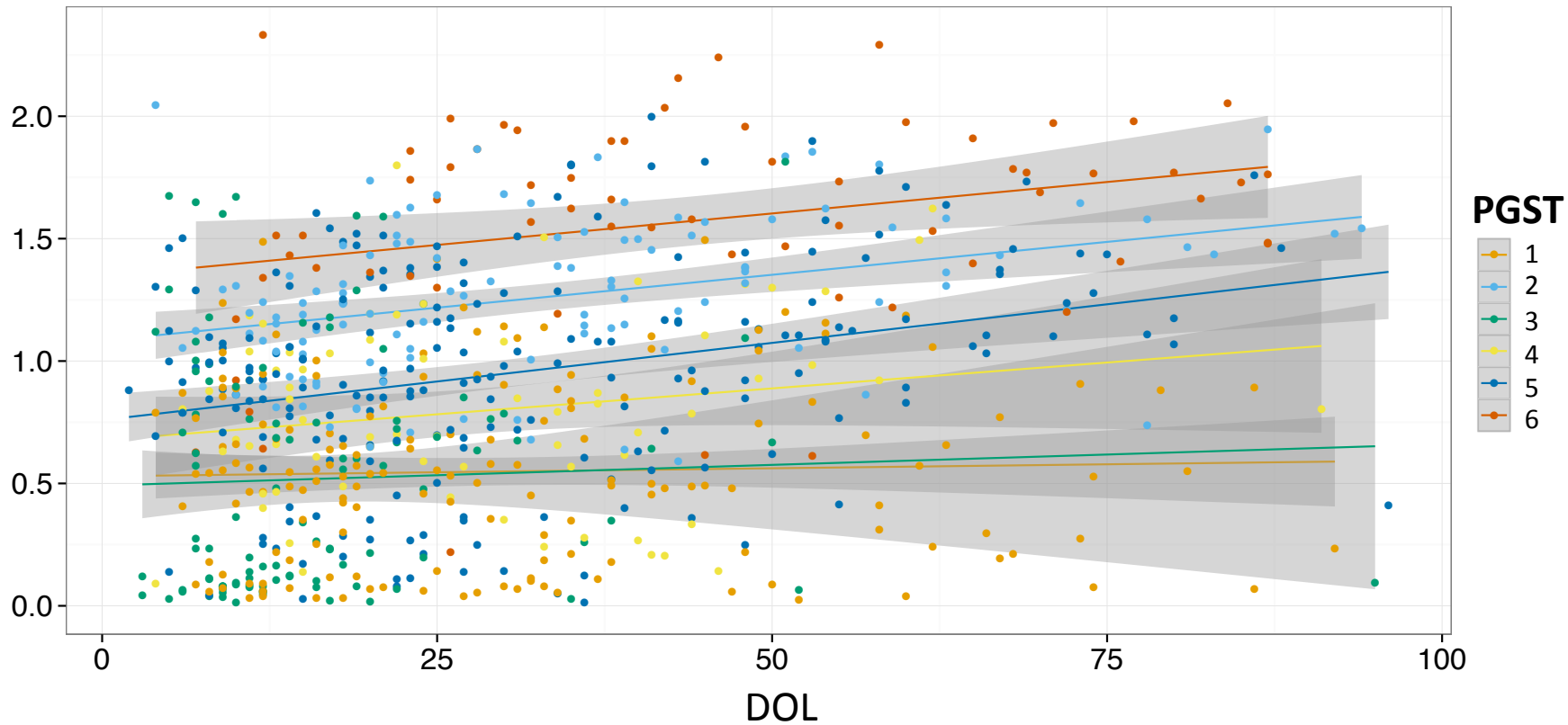

Supplement: Additional file 6: Figure S5. — Shannon diversity indices overtime of all samples grouped according to preterm community type (PGCT). Grey shading indicates the 95% confidence interval of the mean. (PDF 64 kb) [file 40168_2016_216_MOESM6_ESM.pdf]

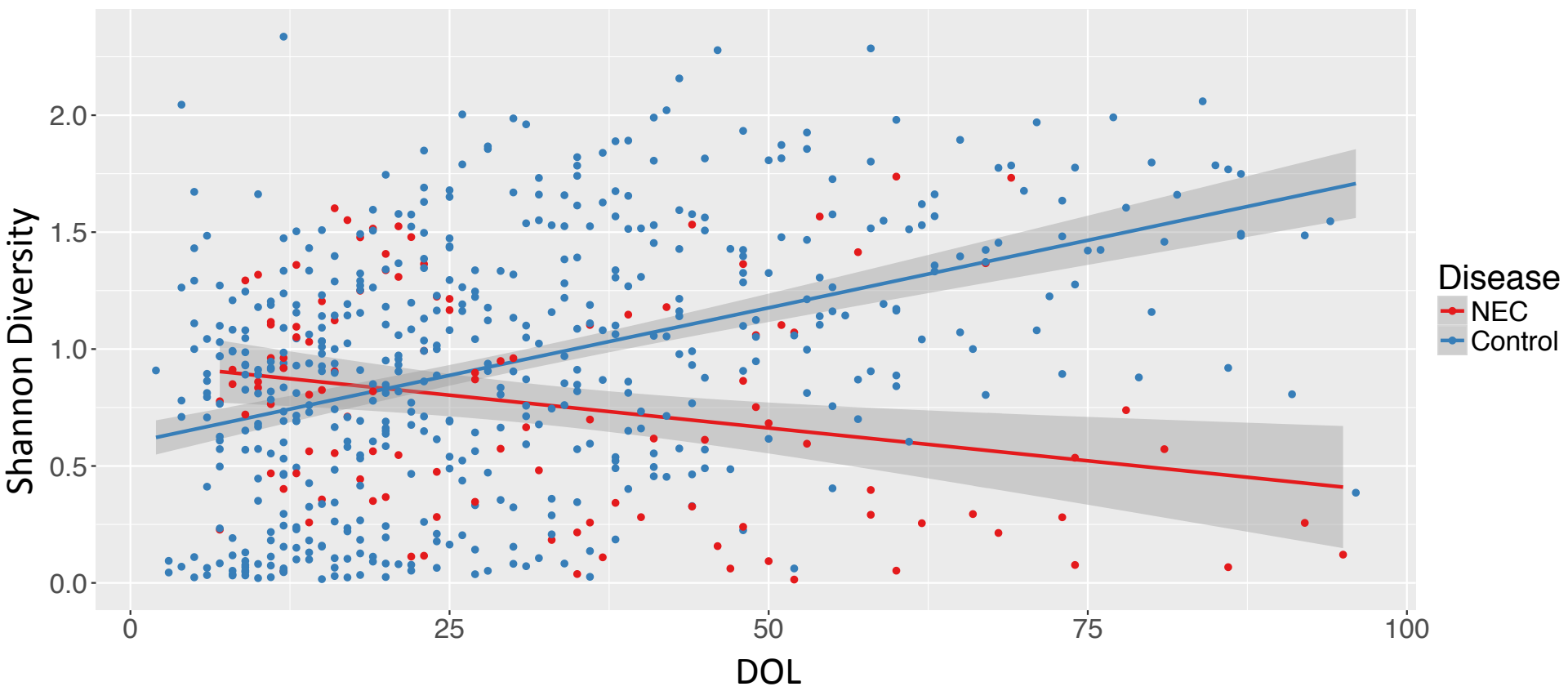

Supplement: Additional file 7: Figure S6. — Shannon diversity indices overtime of all samples grouped according to patient status. Grey shading indicates the 95% confidence interval of the mean. (PDF 40 kb) [file 40168_2016_216_MOESM7_ESM.pdf]

Shannon Diversity

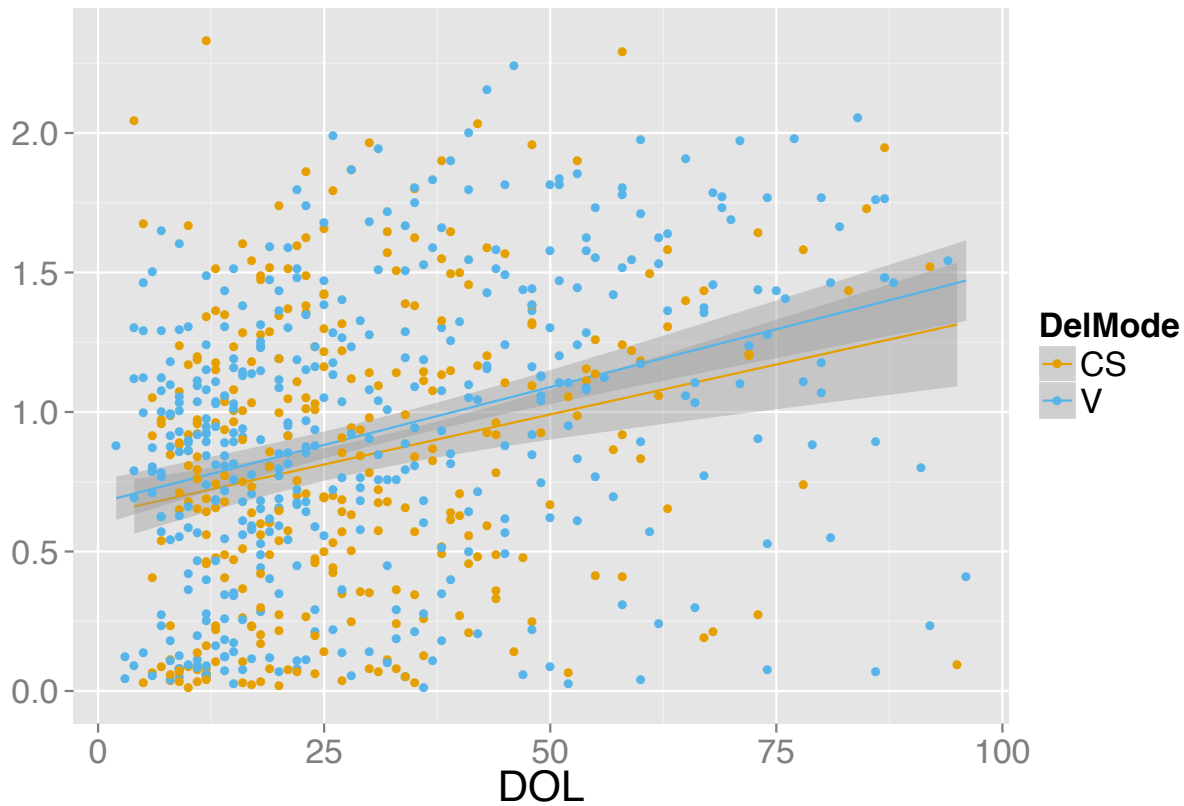

Supplement: Additional file 8: Figure S7. — Shannon diversity indices overtime in infants born by vaginal (V) or caesarean section (CS) delivery. Grey shading indicates the 95% confidence interval of the mean. (PDF 44 kb) [file 40168_2016_216_MOESM8_ESM.pdf]

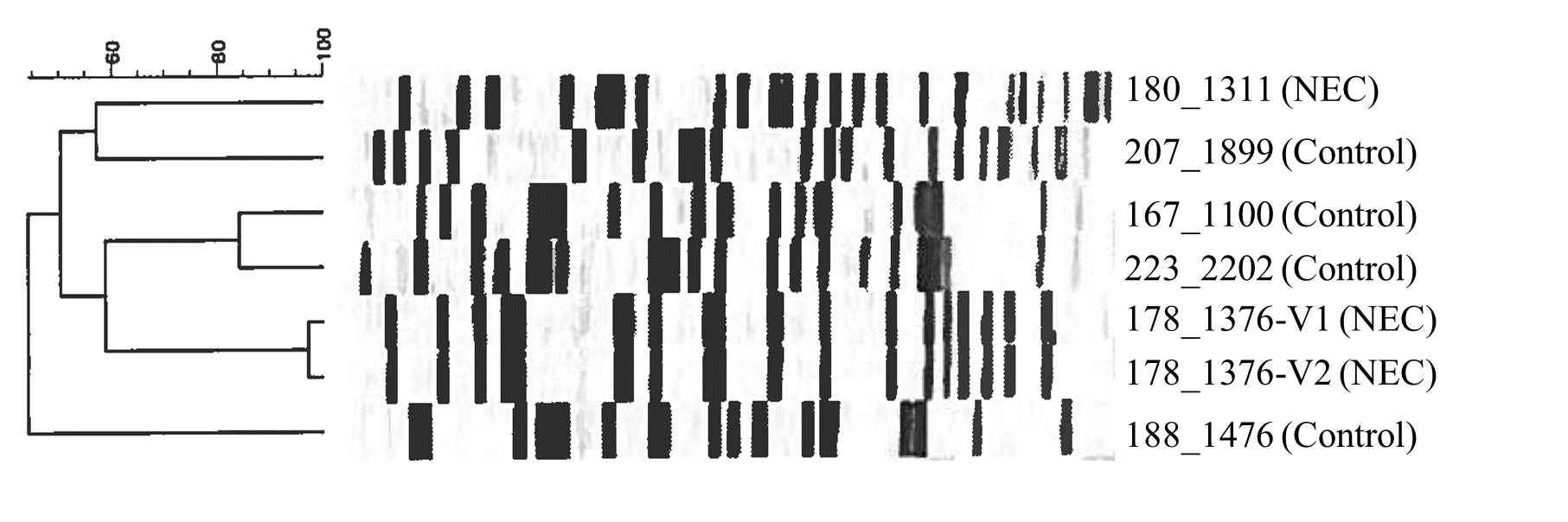

Supplement: Additional file 10: Figure S9. — Pulse field gel electrophoresis (PFGE) of two E. coli isolates from NEC patients and 4 E. coli isolates from control patients. Two isolates from samples 1376 (patient 178) were analysed for confirmatory purposes. (TIF 655 kb) [file 40168_2016_216_MOESM10_ESM.tif]
