# Supplementary material for: Temporal bacterial and metabolic development of the preterm gut reveals specific signatures in health and disease
Source: Microbiome. 2016 Dec 29;4:67. doi: 10.1186/s40168-016-0216-8 (PMC5200962; doi:10.1186/s40168-016-0216-8)
Supplement: Additional file 2: Table S1. — Table of antibiotic information for patients diagnosed with NEC. (DOCX 21 kb) [file 40168_2016_216_MOESM2_ESM.docx]

**Table S1 – Table of antibiotic information for patients with NEC and/or sepsis**

| Patient Number | Abx start (days) | Abx 1 (days of usage) | Abx 2 (days of usage) | Abx 3 (days of usage) | Abx 4 (days of usage) | Abx 5 (days of usage) |
| --- | --- | --- | --- | --- | --- | --- |
| 180 | 0 | P (2) | G (2) |  |  |  |
|  | 15 | A (3) | F (3) | G (3) |  |  |
|  | 17 | T (9) |  |  |  |  |
|  | 19 | M (18) |  |  |  |  |
|  | 28 | Me (10) | G (7) |  |  |  |
|  | 43 | M (2) | V (2) | C (2) |  |  |
| 163 | 0 | P (2) | G (2) |  |  |  |
|  | 8 | M (7) | V (7) | C (7) |  |  |
|  | 20 | A (2) | F (2) | G (2) |  |  |
|  | 22 | V (3) | C (5) |  |  |  |
|  | 42 | V (7) | M (7) | C (7) |  |  |
|  | 69 | V (2) | C (2) |  |  |  |
|  | 77 | V (2) | C (2) | M (2) |  |  |
|  | 86 | F (2) | G (2) |  |  |  |
| 161 | 0 | P (2) | G (2) |  |  |  |
|  | 6 | C (7) | V (7) | M (7) |  |  |
|  | 19 | C (6) | V (6) |  |  |  |
|  | 20 | M (5) |  |  |  |  |
|  | 27 | M (10) | A (10) | G (10) |  |  |
|  | 61 | A (5) |  |  |  |  |
| 199 | 0 | P (2) | G (2) |  |  |  |
|  | 23 | A (4) | F (4) | G (6) |  |  |
|  | 25 | M (10) |  |  |  |  |
|  | 27 | V (8) | C (8) |  |  |  |
|  | 67 | F (6) |  |  |  |  |
|  | 74 | C (2) | V (2) |  |  |  |
| 171 | 0 | P (3) | G (3) |  |  |  |
|  | 9 | V (2) | C (2) |  |  |  |
|  | 13 | A (3) | G (3) | F (3) |  |  |
|  | 17 | A (4) | G (5) | F (4) |  |  |
|  | 19 | M (8) |  |  |  |  |
|  | 21 | V (6) | C (6) |  |  |  |
| 174 | 0 | P (5) | G (5) |  |  |  |
|  | 5 | V (2) | C (2) |  |  |  |
|  | 25 | A (2) | M (7) | C (7) |  |  |
|  | 41 | V (2) |  |  |  |  |
| 139 | 0 | P (2) | G (2) |  |  |  |
|  | 28 | F (4) | A (4) | G (4) | M (4) |  |

A – Amoxicillin, C – Ceftazidine, F – Flucloxacillin, G – Gentamicin, L – Linezolid, M – Metronidazole, Me – Meropenem, P – Penicillin, T – Tazocin, V - Vancomycin
