# Supplementary material for: Temporal bacterial and metabolic development of the preterm gut reveals specific signatures in health and disease
Source: Microbiome. 2016 Dec 29;4:67. doi: 10.1186/s40168-016-0216-8 (PMC5200962; doi:10.1186/s40168-016-0216-8)
Supplement: Additional file 9: Figure S8. — Comparison of the relative abundance of Gammaproteobacteria between preNEC and controls. Only the preNEC samples included from NEC infants and only samples up to day 28 of life in controls. A) Regression analysis over day of life (DOL). B) Box plots. (PDF 52 kb) [file 40168_2016_216_MOESM9_ESM.pdf]

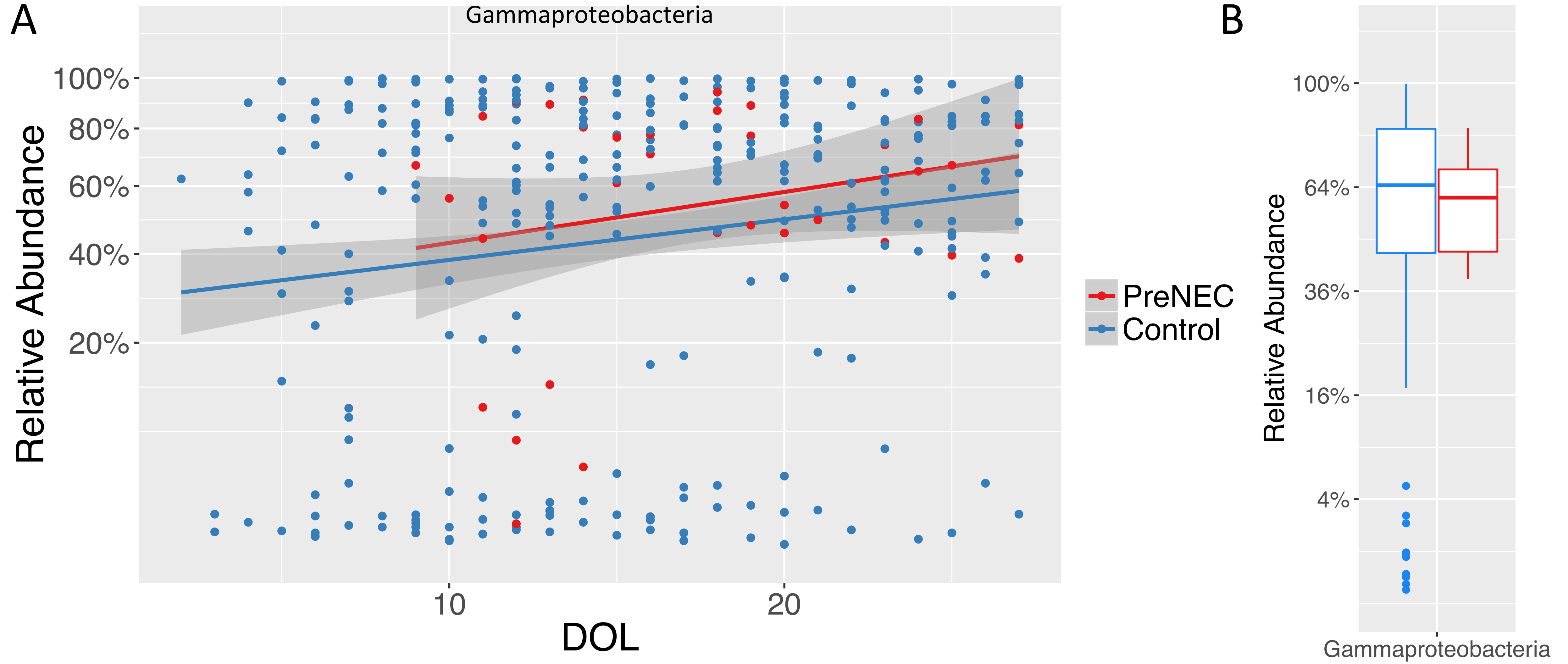

A
Gammaproteobacteria
100%
80%
60%
40%
20%
Relative Abundance
10
20
DOL
PreNEC
Control
B
100%
64%
36%
16%
4%
Relative Abundance
Gammaproteobacteria
